# A risk-predictive model for invasive pulmonary aspergillosis in patients with acute exacerbation of chronic obstructive pulmonary disease

**DOI:** 10.1186/s12931-021-01771-3

**Published:** 2021-06-09

**Authors:** Yu Gu, Xianping Ye, Yuxiu Liu, Yu Wang, Kunlu Shen, Jinjin Zhong, Bilin Chen, Xin Su

**Affiliations:** 1 Department of Respiratory and Critical Care Medicine, Jinling Hospital, Nanjing Medical University, Nanjing, 210002 China; 2 Department of Medical Statistics, The First School of Clinical Medicine, Southern Medical University, Nanjing, 210002 China; 3 Department of Respiratory and Critical Care Medicine, Jinling Hospital, Medical School of Nanjing University, Nanjing, 210002 China; 4 Department of Respiratory and Critical Care Medicine, The First School of Clinical Medicine, Southern Medical University, Nanjing, 210002 China

**Keywords:** IPA, AECOPD, Risk factors, Nomogram, Model

## Abstract

**Objectives:**

Invasive pulmonary aspergillosis (IPA) is increasingly reported in chronic obstructive pulmonary disease (COPD) patients. These patients often have poor clinical outcomes. Early recognition of IPA in COPD is always challenging. We aimed to develop and validate a risk model using readily available clinical parameters to predict IPA for acute exacerbation of COPD (AECOPD) patients.

**Methods:**

We performed a retrospective cohort study. AECOPD patients who were admitted to Jinling Hospital between January 2012 and December 2017 were included. 880 AECOPD patients were randomly divided into the training set (70%, n = 616) and validation set (30%, n = 264). A nomogram model was developed using multivariate logistic regression from training set. The discrimination and calibration of model were validated internally. Decision curve analyses assessed the clinical utility of the nomogram.

**Results:**

The incidence of IPA in hospitalized AECOPD patients was 9.6% in the training set (59 cases of IPA) and 9.1% in the validation set (24 cases of IPA), respectively. The nomogram model consisted of independent factors associated with IPA included lung function GOLD III–IV, use of broad-spectrum antibiotic over 10 days in the last month, oral or intravenous corticosteroids (prednisone) over 265 mg in the last 3 months and serum albumin < 30 g/L. The model performed good discrimination and calibration in validation set (c-statistic, 0.79 [95%CI 0.68–0.90]). The 95%CI region of calibration belt did not cross the 45-degree diagonal bisector line (*P* = 0.887).

**Conclusion:**

The simple risk predictive model for earlier recognition of IPA is useful in hospitalized AECOPD patients.

**Supplementary Information:**

The online version contains supplementary material available at 10.1186/s12931-021-01771-3.

## Background

Nowadays, chronic obstructive pulmonary disease (COPD) has been widely recognized as a major risk factor for invasive pulmonary aspergillosis (IPA) [1]. The current mortality rate might be as high as 100% in untreated patients [2, 3]. Delayed diagnosis or delayed antifungal therapy are associated with increased mortality in patients with IPA [4, 5]. Early recognition of IPA represents an opportunity to improve clinical outcome. In fact, the diagnosis of IPA in COPD patients is difficult. One of the important reasons is that the clinical manifestations and imaging presentations are not specific. On the other hand, the current laboratory diagnostic tests are not very sensitive for non-neutropenic population including COPD patients, leading to delays in diagnosis and treatment of IPA [4, 6, 7].

Combination of risk factors, clinical manifestations, and laboratory tests results together, is the current strategy for IPA diagnosis. Fully revealing risk factors of IPA in COPD patients is important to help clinicians identify infection [8]. In recent years, it has been revealed that worse lung function (GOLD III or IV) and systematic usage of corticosteroid play a significant role in the development of IPA [1]. Studies have also shown that COPD patients who were admitted to ICU, had chronic heart failure, receiving antibiotic treatment longer than 10 days in the past 3 months are independent predictors of IPA in COPD patients [3, 9]. The identification of one or more predisposing conditions would be critical to trigger further diagnostic exploration, which is a benefit to the early diagnosis and treatment.

There are multiple risk factors of IPA in COPD patients. But, few studies focused on the weight of each related risk factors. There is no Risk-Predictive scoring model of IPA currently. Therefore, it is necessary to further investigate the risk factors of IPA in COPD patients. It is of great importance for early diagnosis and treatment.

This study aims to identify the risk factors for IPA in hospitalized acute exacerbation of COPD (AECOPD) patients. Then, we develop and validate a risk-prediction model for rapid recognition and appropriate empirical antifungal treatment in severe AECOPD patients, especially in source limited hospitals.

## Methods

### Data source

This retrospective single-center study was based on the clinical records of AECOPD patients retrieved from the Department of Respiratory and Critical Care Medicine of Jinling Hospital from January 2012 to December 2017.

*Training set and validation set.* AECOPD patients fulfill with study design were enrolled into our study. We randomly assigned 70% cases into training set, and 30% cases into internal validation set.

### Participants

We included patients with AECOPD needing hospitalization. The exclusion criteria were as follows: (1) Patients suffering from neutropenia (peripheral blood absolute neutrophils count less than 0.5×109/L) or with hematological malignancy; (2) patients admitted to hospital without AECOPD; (3) patients with insufficient information. The diagnosis of AECOPD was based on the Global Initiative for Chronic Obstructive Lung Disease (GOLD) guidelines [10]. Bulpa criteria was applied to IPA diagnosis [1]. Therefore, Proven IPA was diagnosed by existence of mycelium and related tissue damage in histopathological examination of lung tissue and was accompanied by any of the following: (1) isolation of *Aspergillus* in the lower respiratory tract (LRT) samples; (2) positive serum *Aspergillus* antigen or antibody; (3) direct molecular immunology or culture methods observed that the mycelium was *Aspergillus* filaments. Probable IPA was diagnosed by the coexistence of host factors (severe COPD patients according to GOLD stage and usually treated by steroids), clinical manifestations (COPD patients had a recent exacerbation of dyspnea and suggestive chest imaging, and poor response to regular treatment) and microbiological evidence (*Aspergillus* isolation in LRT sample or two consecutive positive serum galactomannan [GM] tests). As for possible IPA, it required host factors but without microbiological evidence. Colonization was defined as isolation of *Aspergillus* in LRT samples without any symptom or new pulmonary infiltrate. In this study, Proven/probable IPA were taken as IPA; possible IPA with positive response to antifungal therapy were also considered as IPA.

### Risk factors

Data were collected from eligible AECOPD patients concerning potential risk factors for IPA. All risk factors were readily accessible from early current and past medical history, including demographic data, comorbidities, pulmonary function, pharmacological history (including oral or intravenous corticosteroids in the last 3 months and use of broad-spectrum antibiotic longer than 10 days in the last month), mechanical ventilation at admission and previous history of acute exacerbation. Detailed data were shown in Table 1.

**Table 1:** Clinical characteristics of AECOPD inpatients in the training set and validation set

	No. (%)*		
Characteristic	Training set (n = 616)	Validation set (n = 264)	P
IPA	59 (9.6)	24 (9.1)	0.821
Age, years			
Mean ± SD	75 ± 10.8	75 ± 10.6	0.936
Gender male	525 (85.2)	222 (84.1)	0.666
Smoking index > 400	113 (18.3)	59 (22.3)	0.170
Comorbidities			
Previous tuberculosis	37 (6)	21 (8)	0.286
Lung cancer	25 (4.1)	17 (6.4)	0.129
Bronchiectasis	8 (1.3)	7(2.7)	0.163
Asthma	18 (2.9)	14 (5.3)	0.084
Lobectomy surgery	13 (2.1)	10 (3.8)	0.153
Other solid tumor	23 (3.7)	13 (4.9)	0.414
Hypertension	297 (48.2)	123 (46.6)	0.659
Diabetes mellitus	94 (15.3)	43 (16.3)	0.700
Congestive heart failure	119 (19.3)	72 (27.3)	0.009
Chronic and acute kidney disease	44 (7.1)	15 (5.7)	0.427
Advanced liver disease	8 (1.3)	1 (0.4)	0.292
Connective tissue disease	13 (2.1)	7 (2.7)	0.622
Laboratory results			
Serum albumin < 30 g/L ^‡^	151 (24.5)	70 (26.5)	0.530
GOLD III–IV^§^	409 (66.4)	177 (67)	0.852
Respiratory failure	129 (20.9)	74 (28)	0.022
Co-infection			
Lung bacterial infection	100 (16.2)	54 (20.5)	0.131
Pulmonary tuberculosis	12 (19.5)	4 (1.6)	0.788
Previous treatment			
Inhale corticosteroids	124 (20.1)	47 (17.8)	0.424
Oral or intravenous corticosteroids	59 (9.6)	36 (13.6)	0.075
Cytotoxic drug utility	2 (0.3)	4 (1.5)	0.070
Broad-spectrum antibiotic > 10 days	45 (7.3)	28 (10.6)	0.104
Invasive ventilator utility	49 (8)	28 (10.6)	0.202
ICU admission 1 month prior	54 (8.8)	39 (14.8)	0.008
Hospital acute exacerbation ≥ 2/year	111 (18)	51 (19.3)	0.649

*IPA* invasive pulmonary aspergillosis, *GOLD* global initiative for chronic obstructive lung disease, *ICU* intensive care unit

^*^Values are presented as numbers and percentages, unless otherwise indicated

^‡^This result was obtained from the hospital admission

^§^The GOLD stage was obtained from the latest pulmonary function test within the last year

### Statistical analysis

Statistical analysis was performed using statistical software SPSS (version 25.0, Chicago, IL, USA) and R software (version 3.5.2). Data were expressed as mean ± standard deviation (SD) or median (interquartile [IQR]) for continuous variables, whereas categorical variables were summarized as counts (percentage). Differences between patients in training and validation set, and between patients with and without IPA in the training set were explored using χ^2^ or Fisher exact test for categorical variables, t test for normally distributed continuous variables and Mann-Whitney U test for abnormally distributed variables. Variables with *P* < 0.05 in univariate analysis of training set were substituted into multivariate analysis. We then implemented multivariate logistic regression analysis based on backward stepwise likelihood-ratio method, setting a *P* value < 0.05 for the inclusion of variables. A nomogram of risk-predictive model for IPA was developed from the regression purposeful variable by library ‘rms’ in R [11]. Patients in the internal validation set were used for assessing the discrimination and calibration of the nomogram. The discriminative ability was measured using the area under the ROC curve (AUC), which known as the c-statistic. Calibration of the model was assessed by comparison of the predicted and observed probability of IPA [12]. The fit of the scoring model was evaluated by the Hosmer–Lemeshow goodness-of-fit test. Decision curve analysis was performed according to van Calster et al. to assess the clinical utility of the nomogram, using the library ‘rmda (risk model decision analysis)’ in R [13]. Unless stated otherwise, a two-tailed *P* value < 0.05 was considered statistically significant.

## Results

We screened 1277 hospitalized patients primarily diagnosed as AECOPD. 397 patients were excluded for the following reasons: neutropenia (n = 1); *Aspergillus* colonization (n = 10); not real AECOPD (n = 298); insufficient clinical data (n = 82). 83 cases were diagnosed as IPA (9.4%), with an average age of 75.2±10.7 (40–101 years old). The training set was consisted of 616 patients (IPA group 59 cases, non-IPA group 557 cases), and the validation set was consisted of 264 patients (IPA group 24 cases, non-IPA group 240 cases). Among all the IPA patients, 13 cases were diagnosed with proven IPA, 60 cases with probable IPA (20 cases with positive culture results of *Aspergillus* in the LRT specimens, 25 cases with two consecutive positive serum GM and 3 cases for positive bronchoalveolar lavage fluid (BALF) GM tests, 10 cases with positive results in both sputum culture and serum GM, 2 cases for positive results in BALF GM tests and the sputum test), and 10 cases with possible IPA all had positive response to antifungal therapy. IPA group in the training set consisted of 8 patients with proven IPA, 42 patients with probable IPA and 9 patients with possible IPA. IPA group in the validation set consisted of 5 patients with proven IPA, 18 patients with probable IPA and 1 patient with possible IPA. The flow chart shows the strategy to identify the participants of the AECOPD cohort (Additional file 1: Figure S1). The demographic and clinical characteristics of patients in the training set and validation set are listed in Table 1. The IPA incidence was comparable between two data sets (9.6% vs 9.1%; *P* = 0.821).

The characteristics of AECOPD patients with and without IPA in training set were summarized in Table 2. There was no difference in each group of co-infection including pulmonary tuberculosis. But, two groups varied from each other in terms of treatment before or after patients admitted to the ward, such as oral or intravenous corticosteroids in the last 3 months, use of broad-spectrum antibiotic longer than 10 days in last month, invasive ventilator utility and ICU admission in previous 30 days. Table 2 showed that IPA group had a significant difference with non-IPA in terms of 8 factors, including hypertension, serum albumin < 30 g/L, lung function GOLD III–IV, use of broad-spectrum antibiotic longer than 10 days in last month and so on.

**Table 2:** Comparison of AECOPD inpatients with and without IPA in the training set

Characteristic	No. (%)^*^		P
	IPA (n = 59)	Non-IPA (n = 557)	
Age, years			
Median (range)	72 (67, 82)	76 (68, 84)	0.08
Male gender	55 (93.2)	470 (84.4)	0.69
Smoking index > 400	16 (27.1)	97 (17.4)	0.07
Comorbidities			
Previous tuberculosis	6 (10.2)	31 (5.6)	0.15
Lung cancer	4 (6.8)	21 (3.8)	0.29
Bronchiectasis	1 (1.7)	7 (1.3)	0.78
Asthma	1 (1.7)	17 (3.1)	0.56
Lobectomy surgery	1 (1.7)	12 (2.2)	0.82
Other solid tumor	0 (0)	23 (4.1)	0.11
Hypertension	21 (35.6)	276 (49.6)	0.04
Diabetes mellitus	7 (11.9)	87 (15.6)	0.44
Congestive heart failure	13 (22)	106 (19)	0.57
Chronic and acute kidney disease	4 (6.8)	40 (7.2)	0.96
Advanced liver disease	2 (3.4)	6 (1.1)	0.14
Connective tissue disease	0 (0)	13 (2.3)	0.24
Laboratory results			
Serum albumin < 30 g/L	29 (49.2)	122 (21.9)	< 0.01
GOLD III–IV	56 (94.9)	353 (63.4)	< 0.01
Respiratory failure	13 (22)	116 (20.8)	0.83
Co-infection			
Lung bacterial infection	10 (16.9)	90 (16.2)	0.88
Pulmonary tuberculosis	0 (0)	12 (2.2)	0.25
Previous treatment			
Inhale corticosteroids	11 (18.6)	113 (20.3)	0.76
Oral or intravenous corticosteroids	18 (30.5)	41 (7.4)	< 0.01
Cytotoxic drug utility	0 (0)	2 (0.4)	0.64
Broad-spectrum antibiotic > 10 days	17 (28.8)	28 (5)	< 0.01
Invasive ventilator utility	9 (15.3)	40 (7.2)	0.03
ICU admission 1 month prior	10 (16.9)	44 (7.9)	0.02
Hospital acute exacerbation ≥ 2/year	20 (33.9)	91 (16.3)	< 0.01

*IPA* invasive pulmonary aspergillosis, *GOLD* global initiative for chronic obstructive lung disease, *ICU* intensive care unit

^*^Values are presented as numbers and percentages, unless otherwise indicated

It’s worth noting that oral or intravenous corticosteroids in the last 3 months before admission was of statistical significance between the IPA and non-IPA groups. Therefore, we examined the cumulative dose of corticosteroids during the patients’ treatment 90 days before their admission and found that 59 out of 616 patients used corticosteroids systematically during the last 90 days. All corticosteroids cumulative dose was calculated on prednisone equivalent basis (0.75 mg of dexamethasone, 4 mg of methylprednisolone or 20 mg of hydrocortisone is equivalent to 5 mg of prednisone). 18 out of 59 patients in IPA group, with a mean cumulative dose of prednisone 718 mg (range, 120–2050 mg), 41 out of 59 patients in non-IPA group, with a mean cumulative dose of prednisone 307 mg (range, 75–1800 mg) in this study. The ROC curve is shown in Additional file 1: Figure S2, the area under the ROC curve was 0.75 (95% CI, 0.62–0.88;P = 0.002). The cut-off cumulative dose of prednisone for the risk of IPA was 265 mg, with a sensitivity of 66.7% and specificity 75.6%.

### Nomogram development

**Fig. 1:**
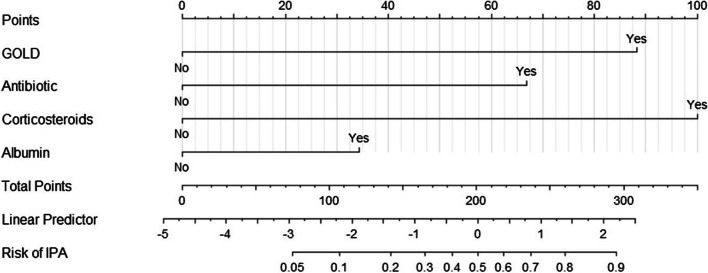
AECOPD inpatients of IPA prediction nomogram. To use the nomogram, the values for each prediction parameter are marked. A vertical line for each mark is drawn downward to determine the points, and total points generate by adding up points of each parameter. A vertical line is followed down to the accompanying line labeled Risk of IPA. The figure on this line indicate the predicted risk that the AECOPD inpatient will develop into IPA. GOLD, pulmonary function as GOLD III–IV. Antibiotic, use utility of broad-spectrum antibiotic over 10 days in the last month. Corticosteroids, Oral or intravenous corticosteroids (equivalent prednisone) ≥ 265 mg last 3 months. Albumin, serum albumin < 30 g/L

The multivariate logistic regression model considered 8 parameters with P value < 0.05, including hypertension, serum albumin < 30 g/L, GOLD III–IV, oral or intravenous corticosteroids, use of broad-spectrum antibiotic longer than 10 days in last month, invasive ventilator utility, ICU admission 1 month previously and hospital acute exacerbation ≥ 2/year. Multivariate logistic analysis showed four independent risk-predictive factors for IPA: lung function GOLD III–IV, oral or intravenous corticosteroids (prednisone) ≥ 265 mg in the last 3 months, use of broad-spectrum antibiotic longer than 10 days in last month, and serum albumin < 30 g/L (Table 3). According to multivariate regression results, the nomogram was generated based on the contributed weights of factors in the training set to calculate the risk of IPA (Fig. 1). In the nomogram, each factor has a related score for its contribution to IPA.

**Table 3:** Multivariate logistic regression for IPA in the training set

Variable	β coefficient	Wald	OR (95%CI)	P
Serum albumin < 30 g/L	0.8	6.7	2.23 (1.22 to 4.1)	0.01
GOLD III–IV	2.06	11.2	7.87 (2.35 to 26.35)	0.001
Dose ≥ 265 mg ^*^	2.34	20.48	10.36 (3.76 to 28.53)	< 0.001
Broad-spectrum antibiotic > 10 days	1.56	16.81	4.77 (2.26 to 10.07)	< 0.001

*OR* odds ratio, *CI* confidence interval, *Ref* reference variable, *GOLD* global initiative for chronic obstructive lung disease

^*^Oral or intravenous corticosteroids (equivalent prednisone) ≥ 265 mg last 3 months

### Discrimination and calibration

**Fig. 2:**
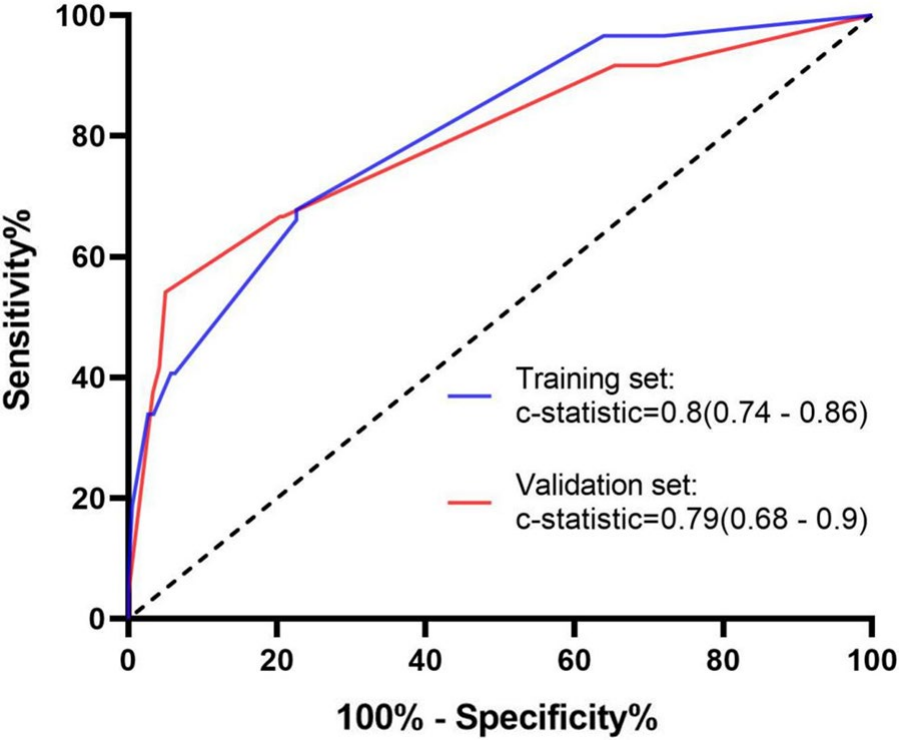
Receiver operating characteristic (ROC) curves showing performance of the prediction model using both the training set and validation set (*P* < 0.0001, vs 0.5)

**Fig. 3:**
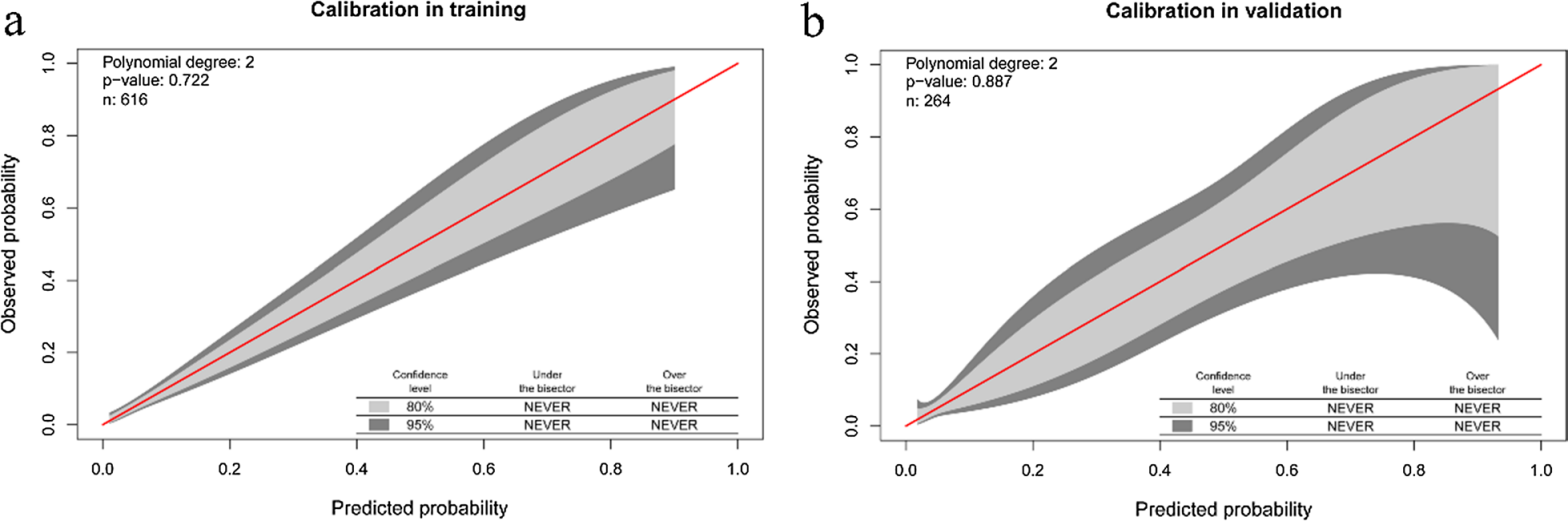
Calibration belt comparing predicted probability of IPA from the nomogram and the observed probability of IPA. The 80%- and 95%-confidence level calibration belt are plotted, in light and dark grey respectively. The red diagonal line is the reference line, indicating the probability of an ideal nomogram. Calibration in training was calculated from training set data, and calibration in validation was calculated from validation set data

The c-statistic of the nomogram was 0.8 (95%CI, 0.74–0.86) in the training set data (Fig. 2, blue curve), and 0.79 (95%CI, 0.68–0.90) in the validation set (Fig. 2, red curve). The calibration belt suggested that the nomogram had strong concordance performance in both the training and validation data sets (Fig. 3a, b). The 95%CI region of GiViTI calibration belt did not cross the 45-degree diagonal bisector line both in two data sets (*P* = 0.722, *P* = 0.887; respectively). The model showed good fit between predicted and observed probabilities because the *P* value for the Hosmer–Lemeshow test was both more than 0.05 in the training set and validation set (*P* = 0.69, *P* = 0.70; respectively).

### Decision curve analysis

Decision curve analysis results of the risk nomogram in the training set and validation set were shown to determine an optimal decision point of the nomogram (Additional file 1: Figure S3). For predicted risk thresholds between 0 and 53%, the nomogram model showed a positive net benefit in two data sets.

## Discussion

In the real-world retrospective study, we developed and internally validated a multivariable model to identify AECOPD patients who are at risk for IPA. The prediction model based on easily accessible clinical parameters. Risk factors of predicting IPA in AECOPD patients comprised albumin, lung function and use of antibiotic and corticosteroids.

IPA in the context of COPD is attracting more and more attention. Current studies have reported that the incidence rate of IPA in COPD stands between 1.3 and 16.13% [7, 14]. This cohort study involved a large population of AECOPD patients, revealing the incidence and mortality of IPA, as well as the risk factors of developing IPA. Furthermore, our study shows a 9.4% incidence of IPA in hospitalized AECOPD patients, which was at a relatively high level. Multiple reasons might lead to such a high incidence. First, our hospital locates in sub-tropical regions of Asia, where environmental fungi grow well due to the temperature and humidity, that might lead to a higher incidence of fungal infection. Second, patients suspected with pulmonary aspergillosis in these regions prefer to transfer to our department, which is a regional center for diagnosis and treatment of pulmonary mycosis.

Patients with AECOPD are often treated with corticosteroids. Current studies prove that corticosteroid is one of the independent risk factors for IPA in COPD [1], but there is no consensus on the cutoff value of accumulative dose of corticosteroids, especially in patients with COPD. Stuck et al, reported that corticosteroids rarely lead to a serious infection in patients with equivalent prednisone dose less than 10 mg/day or a cumulative dose less than 700 mg [15]. The EORTC/MSG Consensus Group definite prolonged use of prednisone at a mean minimum dose of 0.3 mg/kg/day for  > 3 weeks as a host factor for invasive fungal disease [16]. However, the study above was not focused on patients with COPD, and whether the cutoff value of cumulative dose of corticosteroids is suitable for COPD patients remained undetermined. Our study showed that the cumulative dose of systemic prednisone over 265mg in the last 3 months may lead to IPA in hospitalized AECOPD patients, with a sensitivity of 66.7% and specificity of 75.6%. Our study indicates that inpatients with AECOPD may develop IPA even in a low dose of steroids. Besides, cases of IPA have even been reported in patients with long term inhaled high dose of steroids [17, 18].

The existing reports believed that early identification of IPA is essential for early IPA diagnosis and timely treatment [19, 20]. In this regard, our study has made some clinical implications in the rapid identification of patients with high-risk IPA. The study also found that combined poor lung function (GOLD III–IV), serum albumin < 30 g/L and use of broad-spectrum antibiotic longer than 10 days in last month are also independent risk factors for IPA in patients with AECOPD. To our best knowledge, we innovate to develop a simple nomogram for predicting risk of IPA in AECOPD patients. The good discriminative and calibrated ability of the predictive model is found in the internal validation cohort.

Three criteria have been proposed for diagnosing IPA. The criteria defined by the European Organization for Research and Treatment of Cancer/Mycosis Study Group (EORTC/MSG) were created for research in immunocompromised patients, but not good for mild immunocompromised or immunocompetent patients (like ICU or COPD patients) [16]. Clinical algorithm for ICU patients with Aspergillus-positive from lower respiratory tract (LRT) specimens demonstrated favorable validity to discriminate colonization from IPA [21]. The entry criterion of Aspergillus isolation from LRT specimens for clinical algorithm would result in missing diagnosis of probable IPA in ICU [22]. Diagnosis of IPA in our study was based on Bulpa criteria, which was proposed for patients with COPD [1]. Comparing with other two criteria, the Bulpa criteria yielded the highest diagnostic rate of probable IPA in critically ill COPD patients admitted to ICU [23]. Although available literature illustrated risk factors such as use of antibiotic or corticosteroids may predict IPA for COPD patients [7, 24], the accurate contribution and complex association of those factors to predict the likelihood of IPA has not been encompassed by the current criteria.

Nomograms are simple and visual prediction models by combining multiple indicators that can diagnose or predict diseases. The nomogram developed in this study may be useful for clinicians in evaluating the risk of COPD patients complicating IPA, further methods for diagnosing IPA will be actively pursued. For example, using the nomogram, an AECOPD patient with lung function of GOLD III–IV, use of broad-spectrum antibiotic over 10 days in the last month, systemic corticosteroids less than 265 mg last 3 months and serum albumin more than 30 g/L, has a 26% predicted risk of IPA. One study showed that even GOLD II patients treated with steroids were at risk of developing IPA, but those patients would be missed according to Bulpa criteria [25]. An AECOPD patient with two or three risk factors in addition to lung function has 8–52% predicted risk of IPA calculated by the nomogram.

According to the EORTC/MSG and Bulpa criteria, IPA is categorized as proven, probable or possible. AECOPD patients with possible IPA who responded to antifungal therapy were included in this study. Possible IPA had no sufficient mycological evidence. They only performed the test of sputum culture and serum GM, while BALF GM and Aspergillus PCR were not performed. Sensitivity of serum GM is notably lower in non-neutropenic than neutropenic patients [26, 27]. Compared to serum GM and LRT Aspergillus isolation, BALF GM had an excellent performance with higher sensitivity in diagnosis of IPA for COPD patients [28]. Meanwhile, clinical records showed possible IPA in our study received antifungal therapy before serum GM test, which would decrease the positive rate of serum GM.

Our study had some limitations. First, this study was a single-center retrospective study and we only did internal validation to evaluate the discrimination and calibration of the scoring model. Multicenter prospective studies should be conducted to externally validate the results. Second, the binary result (positive/negative) used to classify COPD co-morbidities cannot reflect the severity of the combined disease. Severity classifying of the comorbidities might add the value of the results.

In conclusion, the nomogram model, which is consisted of four independent risk factors for IPA, may empower clinicians and AECOPD patients with earlier, more accurate information regarding the risk of IPA. Further studies are needed to validate the application of the nomogram in clinical practice to determine whether IPA can be better predicted.

## Supplementary Information

**Additional file 1: Figure S1.** Flow Chart for Training and Validation Patients Screening. **Figure S2.** Receiver operating characteristic (ROC) curve for cumulative dose of prednisone for the risk of IPA. Area under the ROC curve was 0.75 (95%CI, 0.62–0.88). P = 0.002. **Figure S3.** Decision curve analysis for the training set and validation set demonstrating the benefit for predicting clinically significant IPA for the nomogram. Dark grey line (All) is the net benefit of providing all AECOPD patients with IPA therapy. Black line (None) is the net benefit of providing no AECOPD patients with IPA therapy. The red curve indicates the net benefit provided by nomogram model in the training set and validation ser data. **Table S1.** TRIPOD Checklist.

## Data Availability

The datasets used and/or analyzed during the current study are available from the corresponding author on reasonable request.

## Abbreviations

IPA: Invasive pulmonary aspergillosis
COPD: Chronic obstructive pulmonary disease
AECOPD: Acute exacerbation of chronic obstructive pulmonary disease
ICU: Intensive care unit
GOLD: Global Initiative for Chronic Obstructive Lung Disease
LRT: Lower respiratory tract
GM: Galactomannan
SD: Standard deviation
IQR: Interquartile
AUC: Area under the ROC curve
BALF: Bronchoalveolar lavage fluid
EORTC/MSG: European Organization for Research and Treatment of Cancer and the Mycoses Study Group Education and Research Consortium
