# Supplementary material for: A risk-predictive model for invasive pulmonary aspergillosis in patients with acute exacerbation of chronic obstructive pulmonary disease
Source: Respir Res. 2021 Jun 9;22:176. doi: 10.1186/s12931-021-01771-3 (PMC8188951; doi:10.1186/s12931-021-01771-3)
Supplement: Supplementary file 1 — Additional file 1: Figure S1. Flow Chart for Training and Validation Patients Screening. Figure S2. Receiver operating characteristic (ROC) curve for cumulative dose of prednisone for the risk of IPA. Area under the ROC curve was 0.75 (95%CI, 0.62–0.88). P = 0.002. Figure S3. Decision curve analysis for the training set and validation set demonstrating the benefit for predicting clinically significant IPA for the nomogram. Dark grey line (All) is the net benefit of providing all AECOPD patients with IPA therapy. Black line (None) is the net benefit of providing no AECOPD patients with IPA therapy. The red curve indicates the net benefit provided by nomogram model in the training set and validation ser data. Table S1. TRIPOD Checklist. [file 12931_2021_1771_MOESM1_ESM.docx]

**Additional Figures and Table**

**Figure S1. Flow Chart for Training and Validation Patients Screening**


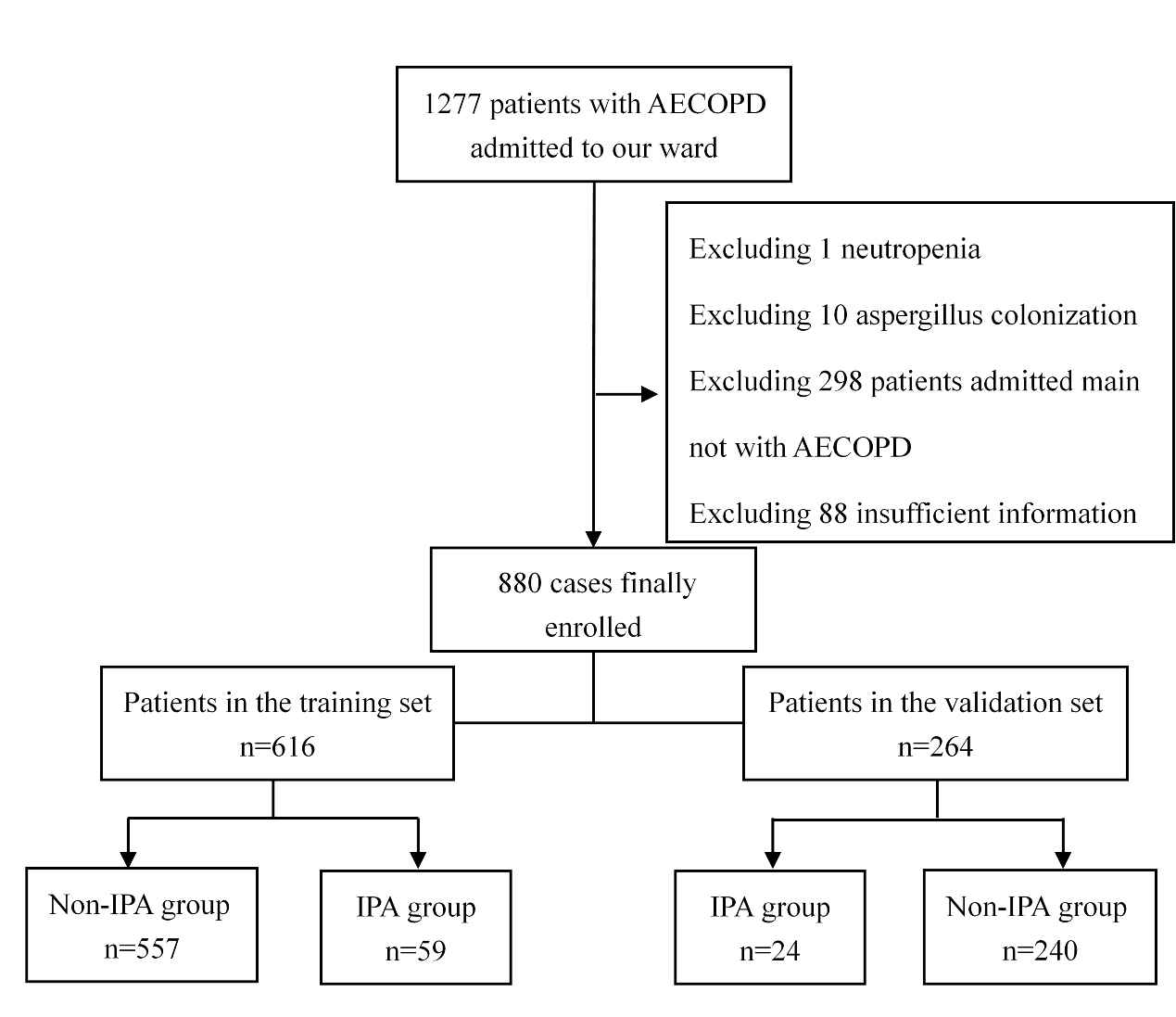


**Figure S2. Receiver operating characteristic (ROC) curve for cumulative dose of prednisone for the risk of IPA.** Area under the ROC curve was 0.75 (95%CI, 0.62-0.88). P=0.002.


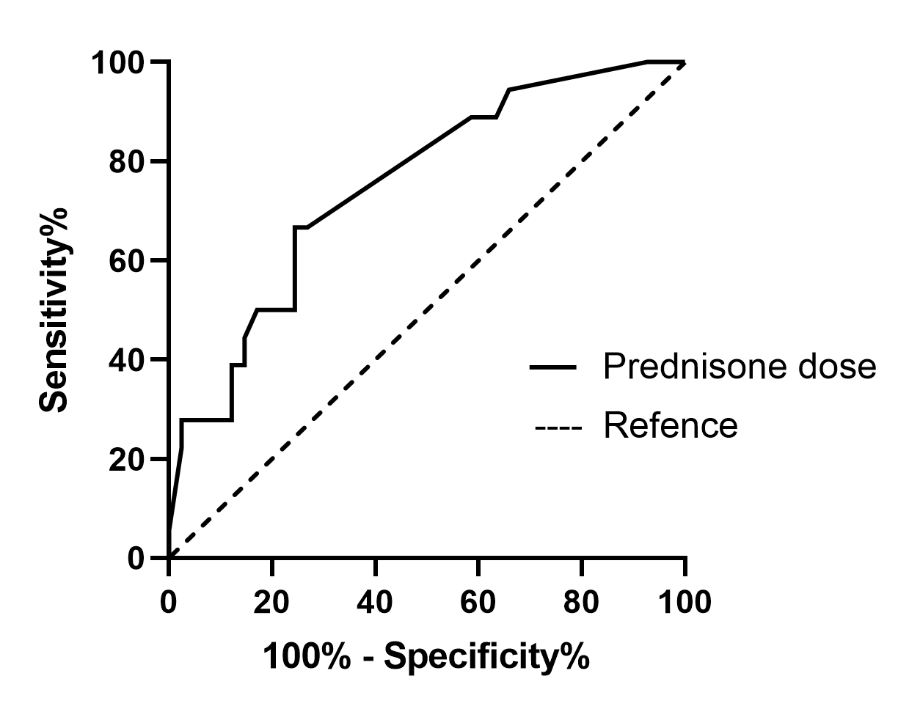


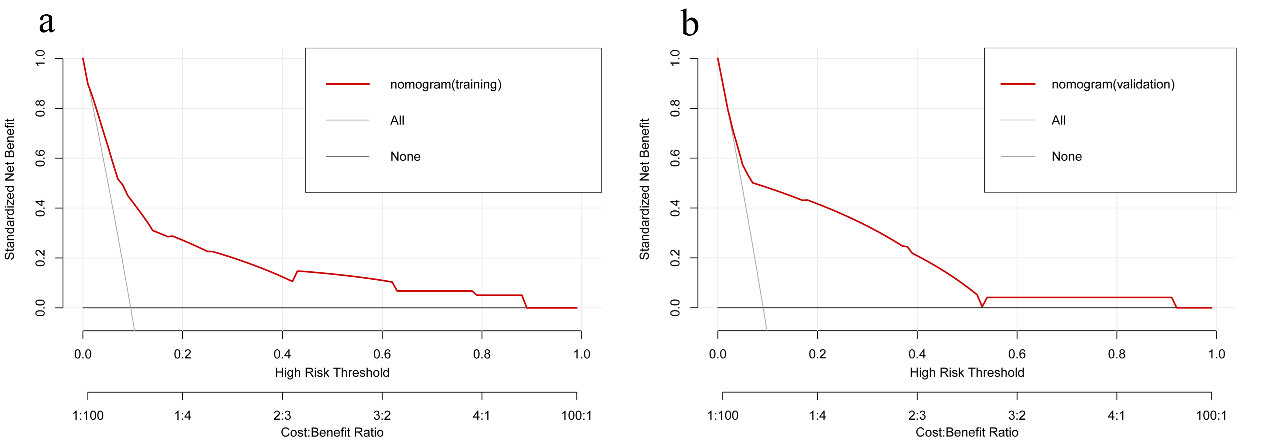


**Figure S3. Decision curve analysis for the training set and validation set demonstrating the benefit for predicting clinically significant IPA for the nomogram.** Dark grey line (All) is the net benefit of providing all AECOPD patients with IPA therapy. Black line (None) is the net benefit of providing no AECOPD patients with IPA therapy. The red curve indicates the net benefit provided by nomogram model in the training set and validation ser data.

**Table S1. TRIPOD Checklist**

| **Section and Topic** | **Item** | **Development or Validation ^*^** | **Checklist Item** | **Page** |
| --- | --- | --- | --- | --- |
| **Title and abstract** |  |  |  |  |
| Title | 1 | D; V | Identify the study as developing and/or validating a multivariable prediction model, the target population, and the outcome to be predicted. | 1 |
| Abstract | 2 | D; V | Provide a summary of objectives, study design, setting, participants, sample size, predictors, outcome, statistical analysis, results, and conclusions. | 2 |
| **Introduction** |  |  |  |  |
| Background and objectives | 3a | D; V | Explain the medical context (including whether diagnostic or prognostic) and rationale for developing or validating the multivariable prediction model, including references to existing models. | 2, 3 |
|  | 3b | D; V | Specify the objectives, including whether the study describes the development or validation of the model or both. | 2, 3 |
| **Methods** |  |  |  |  |
| Source of data | 4a | D; V | Describe the study design or source of data (eg, randomized trial, cohort, or registry data), separately for the development and validation data sets, if applicable. | 3 |
|  | 4b | D; V | Specify the key study dates, including start of accrual; end of accrual; and, if applicable, end of follow-up. | 3 |
| Participants | 5a | D; V | Specify key elements of the study setting (eg, primary care, secondary care, general population) including number and location of centers. | 4 |
|  | 5b | D; V | Describe eligibility criteria for participants. | 4 |
|  | 5c | D; V | Give details of treatments received, if relevant. | 4 |
| Outcome | 6a | D; V | Clearly define the outcome that is predicted by the prediction model, including how and when assessed. | 4,5 |
|  | 6b | D; V | Report any actions to blind assessment of the outcome to be predicted. | NA |
| Predictors | 7a | D; V | Clearly define all predictors used in developing or validating the multivariable prediction model, including how and when they were measured. | 4,5 |
|  | 7b | D; V | Report any actions to blind assessment of predictors for the outcome and other predictors. | NA |
| Sample size | 8 | D; V | Explain how the study size was arrived at. | 4 |
| Missing data | 9 | D; V | Describe how missing data were handled (eg, complete-case analysis, single imputation, multiple imputation) with details of any imputation method. | 4,5 |
| Statistical analysis methods | 10a | D | Describe how predictors were handled in the analyses. | 5 |
|  | 10b | D | Specify type of model, all model-building procedures (including any predictor selection), and method for internal validation. | 5 |
|  | 10c | V | For validation, describe how the predictions were calculated. | 5 |
|  | 10d | D; V | Specify all measures used to assess model performance and, if relevant, to compare multiple models. | 5 |
|  | 10e | V | Describe any model updating (eg, recalibration) arising from the validation, if done. | NA |
| Risk groups | 11 | D; V | Provide details on how risk groups were created, if done. | NA |
| Development *v* validation | 12 | V | For validation, identify any differences from the development data in setting, eligibility criteria, outcome, and predictors. | 5 |
| **Results** |  |  |  |  |
| Participants | 13a | D; V | Describe the flow of participants through the study, including the number of participants with and without the outcome and, if applicable, a summary of the follow-up time. A diagram may be helpful. | 5,6, FigS1 |
|  | 13b | D; V | Describe the characteristics of the participants (basic demographics, clinical features, available predictors), including the number of participants with missing data for predictors and outcome. | 5,6, Table1 |
|  | 13c | V | For validation, show a comparison with the development data of the distribution of important variables (demographics, predictors, and outcome). | 5,6, Table2 |
| Model development | 14a | D | Specify the number of participants and outcome events in each analysis. | 7 |
|  | 14b | D | If done, report the unadjusted association between each candidate predictor and outcome. | 7 |
| Model specification | 15a | D | Present the full prediction model to allow predictions for individuals (ie, all regression coefficients, and model intercept or baseline survival at a given time point). | Table3 |
|  | 15b | D | Explain how to the use the prediction model. |  |
| Model performance | 16 | D; V | Report performance measures (with CIs) for the prediction model. | 7, Figure2 |
| Model updating | 17 | V | If done, report the results from any model updating (ie, model specification, model performance). | NA |
| **Discussion** |  |  |  |  |
| Limitations | 18 | D; V | Discuss any limitations of the study (eg, nonrepresentative sample, few events per predictor, missing data). | 9 |
| Interpretation | 19a | V | For validation, discuss the results with reference to performance in the development data and any other validation data. | 8 |
|  | 19b | D; V | Give an overall interpretation of the results, considering objectives, limitations, results from similar studies, and other relevant evidence. | 8 |
| Implications | 20 | Implications | Discuss the potential clinical use of the model and implications for future research. | 8 |
| **Other information** |  |  |  |  |
| Supplementary information | 21 | D; V | Provide information about the availability of supplementary resources, such as study protocol, Web calculator, and data sets. | 10 |
| Funding | 22 | D; V | Give the source of funding and the role of the funders for the current study. | 9,10 |

Abbreviations: TRIPOD, Transparent Reporting of a Multivariable Prediction Model for Individual Prognosis or Diagnosis; NA, not applicable.

^*^ Items relevant only to the development of a prediction model are denoted by D; items relating solely to a validation of a prediction model are denoted by V; and items relating to both are denoted D; V. We recommend using the TRIPOD Checklist in conjunction with the TRIPOD Explanation and Elaboration document.
